# Oral hygiene practices and their sociodemographic correlates among adults in Burkina Faso: results from the First National Survey

**DOI:** 10.1186/s12903-022-02118-0

**Published:** 2022-03-23

**Authors:** Jeoffray Diendéré, Seydou Ouattara, Jean Kaboré, Ibrahim Traoré, Augustin Nawidimbasba Zeba, Séni Kouanda

**Affiliations:** 1 Research Institute for Health Sciences (IRSS), 399 Avenue de la Liberté, 01 BP 545, Bobo-Dioulasso, Ouagadougou Burkina Faso; 2 Université Joseph Ki-Zerbo, Ouagadougou, Burkina Faso; 3 Institut des Sciences de la Santé (INSSA), Bobo-Dioulasso, Burkina Faso; 4 Research Institute for Health Sciences (IRSS) / Institut Africain de Santé Publique (IAPS), Ouagadougou, Burkina Faso

**Keywords:** Oral health, Oral hygiene practices, Sociodemographic factors, Education, WHO-STEPS, Burkina Faso

## Abstract

**Background:**

Sociodemographic parameters are the driving determinants of oral hygiene practices. This study aims to describe oral hygiene practices and associated sociodemographic factors in the Burkinabè population using the first nationally representative data.

**Methods:**

This descriptive, cross-sectional study included 4677 adults through multistage cluster sampling performed during the first WHO STEPS survey conducted in 2013 in Burkina Faso. The practices we considered were the frequencies of tooth cleaning, fluoridated toothpaste use and dentist visits within the last six months. Sociodemographic variables and oral hygiene practices were described, and the first variables were used as the explanatory variables for the seconds in the multivariable analyses.

**Results:**

Individuals who cleaned teeth at least once a day represented 82.8% and at least twice a day represented 31.4%; 25.6% used fluoridated toothpaste and 2.1% visited a dentist. With the highest odds ratio, only being educated was a favourable factor for each oral hygiene practice. Living in an urban area or being a younger adult were favourable factors for cleaning teeth at least twice a day or the use of a fluoridated paste. Female gender applied more to regular tooth cleaning, as well as to dentist visits.

**Conclusion:**

Cleaning teeth at least once a day was common among Burkinabè, while cleaning at least twice a day, the use of fluoridated paste or dentist visits were infrequent. Education was the key favourable determinant for healthy oral hygiene practices, and improving oral health literacy interventions through basic health education should be promoted.

## Background

Chronic diseases are a growing burden to people, health-care systems and societies, and their rapid increase (in burden) is particularly prevalent in low- and middle-income countries (LMICs) [1]. Oral disorders have been ranked among the three leading level 3 causes in terms of incident cases since 1990 [2]. Good oral hygiene practices are efficient in preventing oral diseases [3], including oral and digestive cancers [4, 5] and cardiovascular risks [3]. Both oral health outcomes and access to oral health services are positively correlated with the number of oral health professionals as dental hygienists [6]. However, sub-Saharan African (SSA) LMICs are a short supply of skilled human resources for oral health [7] with wide socioeconomic inequalities in this sector [8], and sociodemographic factors are the crucial determinants for oral hygiene practices or health [9, 10].

The World Health Organization (WHO) mentioned the need for the vast majority of countries to establish a surveillance system for measuring progress in the control of oral disease and promotion of oral health [11]. The standardized tool designed by this health organization for the surveillance of noncommunicable diseases in LMICs, the *stepwise approach to surveillance (WHO STEPS)*, includes a specific section on oral health status and related behaviours [12]. This section specifically explores oral hygiene practices such as the frequencies of tooth cleaning, the use of fluoridated toothpaste and visits to dentists. The first survey using this tool was performed in 2013 in Burkina Faso. In this low-income country, population-based studies on oral health or hygiene are scarce. Nevertheless, among participants living in the East of the country, dental plaque and calculus have been reported in 53% and 80% of adults aged 25–54 years (y) respectively [13]. In 2018, there were only 28 dental surgeons in the country [14]. The objective of our purpose was to describe oral hygiene practices and associated sociodemographic factors among Burkinabè adults using nationally representative data provided by the first WHO STEPS survey.

## Methods

### Study design

A secondary cross-sectional analysis was performed using data from the first WHO STEPS [12] survey conducted in 2013 in Burkina Faso. This study tool is a recommended for surveillance of chronic diseases and their risk factors. The survey is a standardized method to collect, analyse and disseminate data. It includes a representative sample of the study population, which allows the results to be generalizable to the entire population.

### Ethics considerations

The protocol of the STEPS survey was approved by the Ethics Committee for Health Research of the Ministry of Health of Burkina Faso (deliberation No: 2012–12092; December 05, 2012). Written informed consent was systematically obtained from each participant in the STEPS survey.

### Sampling

The nationally representative sample size was calculated to be 4800 adults aged 25–64 years, according to the WHO STEPS methodology [12]. This sample size (4800) considered the estimates by age group and sex and achieved sufficient accuracy by weighting the numbers of age groups for each gender. It was also weighted to ensure representativeness with regard to the living environment.

### Data collection and variables of interest

Data were collected from a questionnaire and direct measurement. Data collection was conducted from 3 September to 24 October 2013. The data were collected using standardized WHO STEPS questionnaires input into laptop computers. Sociodemographic information was recorded via face-to-face interviews in the language spoken by the participant. Participants’ demographic variables included living environment, gender, age, marital status, education level, and occupation. The oral hygiene practices were the frequencies of tooth cleaning, the use of fluoridated toothpaste and visits to the dentist.

### Individuals included for our secondary analyses

We studied variables of individuals with complete data with regard to sociodemographic parameters and hygiene practices specified above. Of the sample of 4800 individuals surveyed, 105 were not eligible; 10 and eight had missing data concerning sociodemographic variables and dentist visits, respectively, and thus, we included 4677 participants for our secondary analyses.

### Statistical analyses

We used StataCorp Stata Statistical Software for Windows (Version 14.0, College Station, Texas, US) to analyse the data. The quantitative variables were expressed as the means ± standard deviations, and the categorical variables were expressed as percentages (%). The chi-squared test was used to compare categorical variables. Logistic regression analysis was performed to identify the sociodemographic factors with each of the four practices. The regression model was determined by backward elimination (i.e., the progressive elimination of nonsignificant factors by decreasing order of significance). For all analyses, a *p* value below 0.05 was considered significant.

## Results

Of the sample studied, nine (0.2%) individuals had no teeth, and among the rest, 82.8% (95% CI 81.6–83.8) had cleaned teeth at least once a day and 31.4% (95% CI 30.1–32.7) at least twice a day. Two hundred forty-seven (5.3%) ignored whether they used fluoridated toothpaste, while 24.2% (95% CI 23.0–254) declared that they used fluoridated toothpaste. Overall, only 2.1% (95% CI 1.7–2.6) visited a dentist in the last six months. Table 1 reports the distribution of oral hygiene practices by sociodemographic characteristics, and Table 2 summarizes the results of stepwise logistic regressions. The factors associated with cleaning teeth at least twice a day were high education level (aOR = 4.0, *p* < 0.001), living in an urban area (aOR = 1.6, *p* < 0.001), professions with regular income (aOR = 1.6, *p* < 0.001), being woman (aOR = 1.4, *p* < 001) or married/cohabiting (aOR = 1.4, *p* < 0.01). Factors associated with the use of a fluoridated paste were a high level of education (aOR = 6.7, *p* < 0.001), living in an urban area (aOR = 2.4, *p* < 0.001) and being young adults (aOR = 1.4, *p* < 0.001). The favourable factors for visiting dentists were a high education level (aOR = 5.7, *p* < 0.001) and being a woman (aOR = 1.6, *p* < 0.05), while being young was a negative factor (aOR = 0.5, *p* < 0.05).

**Table 1:** Distribution of oral hygiene practices by sociodemographic characteristics

Socio-demographic characteristics	Oral hygiene practices				
	N (un-weighted %)	Visiting dentist within the last 6 months	Cleaning teeth at least once a day % (95% CI)	Cleaning teeth at least twice a day	Use of fluoridated toothpaste
	N = 4677	N = 4677	N = 4668^†^	N = 4668^†^	N = 4421^‡^
Age ranges (in years)					
25–30	1306 (27.9)	1.7 (1.1–2.5)	85.8 (83.8–87.7)	36.4 (33.8–39.0)	28.3 (25.8–30.9)
30–44	1988 (42.5)	2.2 (1.6–2.9)	85.1 (83.4–86.6)	31.7 (29.6–33.8)	27.4 (25.4–29.5)
45–64	1383 (29.6)	2.5 (1.8–3.5)	76.4 (74.1–78.6)	26.2 (23.9–28.6)	20.1 (17.9–22.5)
*p* value		NS	***	***	***
Gender					
Men	2245 (48.0)	1.8 (1.3–2.4)	83.1 (81.5–84.6)	29.9 (28.0–31.8)	29.0 (27.1–31.0)
Women	2432 (52.0)	2.5 (1.9–3.2)	82.4 (80.8–83.9)	32.8 (30.9–34.7)	22.4 (20.7–24.2)
*p* value		NS	NS	*	***
Residence area					
Rural	3734 (79.8)	1.6 (1.3–2.1)	80.3 (79.0–81.6)	28.3 (26.8–29.7)	19.6 (18.3–21.0)
Urban	943 (20.2)	4.1 (3.0–5.6)	92.3 (90.4–93.9)	43.7 (40.5–46.9)	48.5 (45.2–51.8)
*p* value		***	***	***	***
Education level					
No formal/no education	3618 (77.4)	1.5 (1.1–1.9)	80.2 (78.8–81.5)	28.3(26.9–29.8)	18.6 (17.3–19.9)
Primary achieved	727 (15.5)	3.2 (2.0–4.7)	88.4 (85.9–90.7)	31.1 (27.7–34.6)	37.2 (33.7–40.9)
Secondary or more	332 (7.1)	6.9 (4.4–10.2)	98.2 (96.1–99.3)	65.4 (60.0–70.5)	72.5 (67.4–77.2)
*p* value		***	***	***	***
Occupation					
Occupation with regular income	3497 (74.8)	1.9 (1.5–2.4)	83.1 (81.8–84.3)	32.5 (31.0–34.1)	25.3 (23.8–26.8)
Job with not regular income	1180 (25.2)	2.8 (1.9–3.9)	81.7 (79.4–83.9)	28.0 (25.5–30.7)	26.5 (24.0–29.2)
*p* value		***	***	***	***
Marital status					
Singles	642 (13.7)	2.5 (1.4–4.0)	82.6 (81.4–83.7)	30.5 (26.9–34.2)	33.9 (30.2–37.9)
Married/cohabiting	4035 (86.3)	2.1 (1.7–2.6)	83.8 (80.7–86.5)	31.5 (30.1–33.0)	24.3 (23.0–25.7)
*p* value		NS	NS	NS	***

^†^Nine individuals without teeth were not included in the analyses; ^‡^Individuals without teeth (nine) or those who did not know if their toothpaste was or was not fluoridated (247) were not included in this analysis; NS: nonsignificant *p* value; *p value < 0.05; **p value < 0.01; ***p value < 0.001

**Table 2:** Sociodemographic correlates of oral hygiene practices in logistic regressions

	Visited a dentist within the last 6 months (N = 4677)		Cleaning teeth at least once a day (N = 4668^†^)		Cleaning teeth at least twice a day (N = 4668^†^)		Use of fluoridated toothpaste (N = 4421^‡^)	
	cOR (95% CI)	aOR (95% CI)	cOR (95% CI)	aOR (95% CI)	cOR (95% CI)	aOR (95% CI)	cOR (95% CI)	aOR (95% CI)
Gender								
Men	1	1	1	1	1	1	1	1
Women	1.4 (0.9–2.1)	1.6 (1.1–2.5)*	0.9 (0.8–1.1)	> 1.0 (0.9–1.2)	> 1.0 (1.1–1.3)*	1.4 (1.2–1.6)***	0.7 (0.6–0.8)***	0.7 (0.6–0.8)***
Place of residence								
Rural	1	1	1	1	1	1	1	1
Urban	2.6 (1.7–3.9)***	1.4 (0.9–2.3)	2.9 (2.3–3.8)***	2.2 (1.6–2.8)***	2.0 (1.7–2.3)***	1.6 (1.3–1.9)***	3.9 (3.3–4.5)***	2.4 (2.0–2.8)***
Age (in years)								
45–64	1	1	1	1	1	1	1	1
30–44	0.9 (0.5–1.3)	0.7 (0.5–1.1)	1.8 (1.5–2.1)***	1.7 (1.4–2.0)***	1.3 (1.1–1.5)**	1.2 (1.1–1.4)*	1.5 (1.3–1.8)***	1.4 (1.2–1.7)***
25–30	0.7 (0.4–1.1)	0.5 (0.3–0.9)*	1.9 (1.5–2.3)***	1.8 (1.4–2.2)***	1.6 (1.4–1.9)***	1.5 (1.3–1.8)***	1.6 (1.3–1.9)***	1.4 (1.2–1.8)***
Education level								
Not formal/not education	1	1	1	1	1	1	1	1
Primary	2.2 (1.3–3.5)**	2.5 (1.5–4.1)***	1.9 (1.5–2.4)***	1.5 (1.1–1.9)**	1.1 (0.9–1.4)	1.1 (0.9–1.2)	2.6 (2.2–3.1)***	1.9 (1.6–2.3)***
Secondary or more	4.9 (3.0–8.1)***	5.7 (3.4–9.4)***	13.4 (6.0–30.3)***	8.1 (3.5–18.4)***	4.8 (3.8–6.1)***	4.0 (3.1–5.2)***	11.6 (8.9–15.0)***	6.7 (5.1–8.9)***
Occupation								
Job with inconstant income/jobless	1	1	1	1	1	1	1	1
Occupation with regular income	(0.4–1.0)	0.8 (0.5–1.3)	1.1 (0.9–1.3)	1.2 (1.1–1.4)*	1.2 (1.1–1.4)**	1.6 (1.4–1.9)***	0.9 (0.8–1.1)	0.9 (0.7–1.1)
Marital status								
Singles	1	1	1	1	1	1	1	1
Married/cohabiting	(0.5–1.4)	1.2 (0.7–2.1)	0.9 (0.7–1.2)	1.2 (0.9–1.5)	1.1 (0.9–1.3)	1.4 (1.2–1.7)**	0.6 (0.5–0.8)***	1.1 (0.9–1.3)

^†^Nine individuals without teeth were not included in the analyses; ^‡^Individuals without teeth (nine) or those who did not know if their toothpaste was fluoridated (247) were not included in this analysis. Stars indicate significant *p* values only, and *p value < 0.05, **p value < 0.01, ***p value < 0.001; aOR: adjusted odds ratio; cOR: crude odds ratio

## Discussion

Healthy oral hygiene practices were infrequently applied by Burkinabè adults, and education was the key determinant for good oral hygiene practices.

### Oral hygiene practices

The majority of Burkinabè cleaned the teeth at least once a day (82.8%), while the recommended number was at least twice a day [15]. Knowledge about oral hygiene was insufficient among Burkinabè [16] and can affect the level of consistent practices. Thus, less than one-third cleaned the teeth at least twice a day, as found in Indian adults (29%) [17]. The use of fluoridated paste was reported by 25.6%, whereas it was reported by 18% in 35-44y old Burkinabè a decade earlier [16]. Even if it appears to be an improvement, personal oral hygiene in the absence of fluorides does not have a benefit in terms of reducing oral diseases such as dental caries [18]. The lower prevalence of those who visited a dentist (2.1%) reflects the difficult access to oral care in low purchasing power areas and the insufficient human resources to face the need in terms of oral health [7, 16, 19]. Tele-dentistry is helpful to facilitate access to oral health care [20, 21] and should be implemented in Burkina Faso.

### Sociodemographic factor correlates with oral hygiene practices

#### High education level

The correlates of high education level with each hygiene practice that we found were close to results reported among Nepalese adults [10], and among students, higher oral health literacy was associated with better oral health practices [22]. This may support why lower education was considered a risk factor for dental plaque or gingivitis [17]. Those with higher education levels usually become more concerned about their own physical or body appearance, including tooth whiteness, and then assume behaviours to this goal. Exposure to oral health education programs during primary school years was found to have positive effects on oral health knowledge and practice [23], and we reported that attending primary school was associated with favourable oral hygiene practices (Table 2). The country would benefit from increasing primary school enrolment and completion rates (respectively at about 89% and 62% in 2019) [24], while integrating simple oral hygiene education modules into the national curriculum as it has been experienced in Bangladesh, Indonesia, Nepal and Tanzania [25]. Since the health literacy is a strong predictor of an individuals' health, health behaviour and health outcomes [26], integrating oral and general health through health literacy practices which would be adapted for the general population, should be implemented [27].

#### Urban residency and young adults

Except for the practice of the dentist visit, other kinds of oral hygiene practices had an urban residency or were younger adults (25–30 y or 30–44 y) as favourable factors, in line with the results of the Nepalese study [10]. In contrast to our study, it also reported that urban residency was a favourable factor for dentist visits (aOR = 1.9, *p* < 0.05) and suggested a higher number of oral health professionals in this country (1400 dentists, one per 20,000 inhabitants), with the highest density of workers in an urban area [28]. Moreover, the specific source of motivations for cleaning teeth or toothpaste selection among young adults was the fear of losing teeth and the whitening feature of teeth [29].

#### Female gender

Concerning associations of the female gender with the good practices of cleaning teeth twice a day [aOR = 1.4 (95% CI 1.2–1.6)], as with visiting dentist [aOR = 1.6 (95% CI 1.1–2.5)], our report was similar to that among Nepalese people with the respective aOR of 1.7 (95% CI 1.1–2.4) and 2.2 (95% CI 1.2–3.8) [10]. In Burkina Faso, women represented approximately 52.0% (as in our representative sample), and married/cohabiting Burkinabè represented 86.3% (Table 1). A simple educational intervention has a positive impact on oral health behaviours in groups [30], and in the framework of family oral hygiene education for Burkinabè societies, women should be placed in a key role. This is quite fitting, especially since being in a group, e.g., married/cohabiting, was also associated with cleaning teeth at least twice a day [aOR = 1.4 (95% CI 1.2–1.7), Table 2].

#### Occupation with regular income

Professions with regular income were a favourable factor for cleaning teeth at least twice a day [aOR = 1.6 (95% CI 1.4–1.9), Table 2]. Professions may determine the income level, and high income among Korean adults was favourable for daily repetitive tooth brushing [31]. A high number of tooth cleanings may imply more financial investments to provide toothpaste, while the share of Burkinabè people living on less than $1.90 a day was 43.7% in 2014 [32]. In contrast to the Australian study reporting that an increased household income improved dental visits [33], we noticed that even Burkinabè with regular income did not have a favourable habit of dentist visits [aOR = 0.8 (95% CI 0.5–1.3) Table 2]. This suggests generalized low purchasing power to face dental care costs in Burkina Faso.

### Limitations

Income variables were not collected, and geographic data were not included in the analyses; thus, we missed specific information on their impact in our multivariable models. While these first nationally representative data from 2013 may no longer reflect the current situation, they provide a relevant baseline that can be compared with future WHO STEPS survey data.

## Conclusion

Cleaning teeth at least once a day was common among Burkinabè but not its regular practice, the use of fluoridated paste or the habit of dentist visits. Education was the key favourable determinant for a good oral hygiene practice. The country should increase primary school enrolment and completion rates while integrating simple oral hygiene education modules into the national curriculum. Family oral hygiene education should be initiated, with women in the key role. Meanwhile, improving access to fluoridated products and the training of dental human resources with their efficient density at the different stages of the national health care system should be undertaken.

## Data Availability

The database of the STEPS survey used for this secondary analysis is available at the Ministry of Health of Burkina Faso. Directed to Dr Brice Bicaba bicababrico78@gmail.com.

## Abbreviations

aOR: Adjusted odds ratios
CI: Confidence interval;
cOR: Crude odds ratio
LMICs: Low- and middle-income countries
NCDs: Noncommunicable diseases
OR: Odds ratio
SSA: Sub-Saharan-African
STEPS: Stepwise approach to surveillance
WHO: World Health Organization
